# A New Palmitoylethanolamide Form Combined with Antioxidant Molecules to Improve Its Effectivess on Neuronal Aging

**DOI:** 10.3390/brainsci10070457

**Published:** 2020-07-17

**Authors:** Vera Morsanuto, Rebecca Galla, Claudio Molinari, Francesca Uberti

**Affiliations:** Laboratory of Physiology, Department of Translational Medicine, University of Piemonte Orientale, Via Solaroli 17, 28100 Novara, Italy; rebecca.galla@uniupo.it (R.G.); claudio.molinari@med.uniupo.it (C.M.)

**Keywords:** palmitoylethanolamide, vitamin D3, lipoic acid, FM-LipoMatrix^®^, neuronal aging

## Abstract

Palmitoylethanolamide is a nutraceutical compound naturally produced in many plants and animal source foods, but the natural form is poorly water-soluble. It has demonstrated an anti-inflammatory role as a neuroprotective mediator, acting on several molecular targets of the central nervous system involved on brain aging process. In healthy adults, palmitoylethanolamide is an endogenous PPAR-α (peroxisome proliferator-activated receptor α) agonist through which it performs anti-inflammatory activity and provides its effects by activating the cannabinoid receptor. The different formulations of palmitoylethanolamide (micronized palmitoylethanolamide, FM-LipoMatrix^®^ palmitoylethanolamide and FM-LipoMatrix^®^ palmitoylethanolamide plus lipoic acid and vitamin D3) were analyzed starting from intestinal barrier, to verify their bioavailability, to in primary astrocytes in which cell viability, reactive oxygen species (ROS) and nitric oxide (NO) production, NFKB activity, MAPK, p53 and PPARα activities were investigated. Additionally, cannabinoid and estrogen receptors were analyzed using the western blot technique. The combination of palmitoylethanolamide in FM-LipoMatrix^®^, lipoic acid and vitamin D3 shows better absorption predicting an improvement on plasma concentration; this formulation also shows a reduction in ROS and NO production and the data show the interaction of palmitoylethanolamide with cannabinoids and estrogen receptors inhibiting neuroinflammatory markers. All these data support the hypothesis of a new potential strategy to restore brain function and slow down brain aging in humans.

## 1. Introduction

Palmitoylethanolamide (PEA), an endogenous fatty acid amide, naturally present in many plants and food sources of animal original, which has recently emerged as a potential nutraceutical supplement [1]. This molecule has been known since 1950 as an anti-inflammatory component of egg yolk. PEA belongs to the N-acylethanolamine (NAE) family of biologically active endogenous lipids, that also includes the ligand of the endogenous cannabinoid receptor anandamide and the satiety factor oleoyletanolamide [2]. Known in Europe since 1970 under the name of impulsin, PEA has been used in preventing viral infections of the respiratory tract. Many studies suggest that PEA may potentially be used in a wide range of therapeutic areas, including pain and neurodegeneration and at the same time is known to be essentially free of adverse effects in humans [2]. Recently, PEA has also demonstrated an analgesic and anti-inflammatory role as a neuroprotective mediator, acting on different molecular targets of both the central nervous system and the immune system. Moreover, PEA is an endogenous molecule in humans and many other living organisms that can play a very important role in maintaining cellular homeostasis and in counteracting exogenous stressful events that can lead to an inflammatory reaction. Among the pharmacological strategies for sensory and affective/cognitive disorders associated with neuropathic pain, PEA showed anti-inflammatory, analgesic, immunomodulatory and neuroprotective effects as well [3,4,5] by restoring glutamatergic transmission homeostasis [3,4,5,6,7,8,9]. For example, PEA demonstrates a neuroprotective action in experimental models of neuroinflammation and neuropathic pain mediated by mast cells and in experimental models of stroke, spinal injury, traumatic brain injury and Parkinson’s disease. The ability of PEA to modulate the protective responses during inflammation derives from its belonging to the homeostatic system that controls inflammation and which is able to counteract the release of inflammatory mediators synthesized de novo by activated mast cells [10]. However, PEA has multiple effects and the ability to act on various cell types, including astrocytes, microglia, and macrophages. In this way, PEA can represent an attractive therapeutic tool for the treatment of pathologies characterized by neurodegeneration and inflammation. The neuroprotective and anti-inflammatory effects of PEA are mainly based on the same molecular mechanism, namely the activation of PPAR-α (peroxisome proliferator-activated receptor α), which reduces the transcription of proinflammatory genes [11]. Since the main mechanism of action of PEA in the central nervous system includes the ability to counteract inflammation, recent findings support the hypothesis of its use in brain aging in both healthy and neurodegenerative conditions. Indeed, neuroinflammation represents a similar target in aging and a wide range of neurological diseases. Normally, healthy brain ageing is also associated with an enhanced level of pro-inflammatory cytokines, which induces a moderate to severe pro-inflammatory condition in the course of neurodegenerative diseases. The increasingly pro-inflammatory environment accelerates brain ageing, contributes to cognitive impairments, and promotes neurodegeneration. In addition, another important factor includes the microglia phenotype, which is primed for pro-inflammatory responses, astrocytes that enhanced expression of glial fibrillary acidic protein and increased secretion of pro-inflammatory factors. Finally, the senescent microglia and astrocytes provide less support for neurons which are responsible for an enhanced pro-inflammatory microenvironment in the brain that may contribute to brain ageing [12]. These observations lead to the introduction of the term “neuroinflammation”, which is a response of the CNS to a changed homeostasis [13]. The actions promoted by the neuroinflammations are classified as: homeostatic (vasodilation and release of cytokines and neurotrophic factors); maladaptive (release of pro-inflammatory factors); neurotoxic (release of pro-inflammatory factors and breakdown of blood–CNS barrier); and anti-inflammatory (release of pro-inflammatory cytokines, neurotrophic factors, neurotransmitters, and cell adhesion molecules. Indeed, the resolution of neuroinflammation is a process that allows to return to homeostasis and in this context the main players are represented by lipid mediators including PEA which is a naturally occurring lipid signaling molecules [13]. For this reason, the modulation of this marker is necessary for designing new neuroprotective strategies [14] since an inflammation that does not resolve is one of the principal contributors to the medical burden in industrialized societies [15]. This hypothesis is based on previous data about ability of PEA to preserve memory in rodent models of Alzheimer’s disease [16] through its interaction with PPAR-𝛾 [17,18,19]. This receptor is expressed both in peripheral metabolic tissues and in various areas of the brain and spinal cord [20], indicating a pleiotropic metabolic and neuroprotective activity. Indeed, PPAR–α, a target for PEA, which is best known for its role in reducing inflammation by decreasing cytokines, pro-inflammatory enzymes and oxidative stress, has a neuroprotective effect in various neurological disorders such as Alzheimer’s disease, Parkinson’s disease, multiple sclerosis, and cerebral ischemia [21]. Recent literature has also identified PPAR–α as a new useful target in a novel approach to treat mood disorders by engaging neurosteroid biosynthesis [22]. PPAR–α activation has been known to be able to modulate stress response and for this reason is an important natural stress marker [23] causing the inhibition of NFKB activity [24]. In healthy adults, PEA, in its role as PPAR–α agonist, significantly increased after clinical stress tests, relative with increased cortisol levels [25]. Another element that deserves attention includes the typical endocannabinoid target in the central nervous system, namely the cannabinoid receptor coupled with G protein type 1 (CB1) [26]. In neural cells, the CB1 and CB2 receptors show opposite expression patterns, with CB1 increasing and CB2 decreasing during neuronal differentiation [27]. It is well known that, during neuronal differentiation, CB1 expression is induced by neurotrophins, such as brain-derived neurotrophic factor (BDNF) and nerve growth factor (NGF). However, the intracellular mechanisms at downstream level of TrkB and TrkA receptors leading to CB1 activity remain largely unknown [27] CB1 receptor levels are associated to increased expression of differentiation markers of various neuronal populations, although the ability of CB1 signaling to act as a molecular switch of neurochemical mechanisms is still unknown. PEA is also an endogenous compound found in most cell types, tissues, and body fluids. It is synthesized and metabolized via various enzymes, namely N-acyl-phosphatidylethanolamine phospholipase D (NAPE-PLD), fatty acid amide hydrolase (FAAH) and/or N-acylethanolamine acid amidase (NAAA), which share the same biosynthetic pathway with the endocannabinoid anandamide (AEA) [28]. Clinical trials proved that exogenous administration of PEA lacks side effects, and since 2008 it has been marketed in different countries as a nutraceutical food supplement [29,30]. Many clinical trials and research papers describe the therapeutic role of PEA inflammation, chronic pain, and neurodegenerative diseases, but its mechanism of action is not yet clarified [31]. PEA was evidenced to act through receptor binding and was initially thought to bind to the cannabinoid 2 receptor (CB2) [32]. However, Further research revealed that PEA, unlike AEA, exhibits only weak binding efficacy on the CB2 receptor, but possesses the capability to affect AEA signaling by acting as a competing substrate [33]. Recent studies showed that PEA provides its effects by activating both CB1 and CB2 receptors. CB1 receptors are distributed in the peripheral and central nervous system being identified in great amounts in the brain cortex, cerebellum, spinal cord, basal ganglia, hippocampus and olfactory areas, owing for the modulatory action of ECS on cognitive function, behaviour, memory, locomotor and emotion activity. On the other hand, CB2 receptors were found in immune system cells (spleen, macrophages). One observed finding is that PEA induces the activation of MAPK, PI/PKB and MEK/ERK signaling pathways promoting an increase in activity of various transcription factors [34]. In this context another important target able to modulate neuroinflammation is ERβ, which is well known for its anti-inflammatory effects on microglia decreasing NO production [35]. It’s important to remember that uncontrolled inflammation is characterized by the overexpression of cytokines, such as TNF and IL, reactive oxygen species (ROS), and other inflammatory mediators (such as NO) able to induce the chronic and degenerative damages [13].

In this context PEA can modulate ERβ protein expression to improve its anti-inflammatory properties. Indeed, the compound has been demonstrated effective in reducing neuroinflammation and neurodegeneration in several in vitro and in vivo models [36].

A drawback of the PEA molecule is its natural poor solubility in water. However, if prepared in micronized or ultramicronized form, its oral administration shows a great efficacy against inflammatory conditions, compared to PEA in native form. Plasma values of PEA show marked daily fluctuations. However, the concentrations found within tissues appear to be higher than the plasma values. Moreover, few data on the pharmacokinetic properties of PEA are available in literature [2]. Plasma PEA concentrations are usually expressed in nM and, as far as its half-life is concerned, it shows a peak concentration at 2 h followed by a decay, as reported by Petrosino [37] who measured the plasma concentration at 0, 2, 4 and 6 h after oral administration of 300 mg of micronized PEA in 10 healthy subjects [37]. The ability to inhibit or modulate the enzymatic metabolic pathway of PEA is therefore a promising complementary therapeutic approach for the treatment of neuro-inflammation. Studies on the tissue distribution of PEA have shown that if this substance is administered in an emulsified form in oil, the amount that reaches the heart and brain increases significantly. This effect is also observed at 24–48 h [38]. The availability of PEA as a (ultra)micronized nutraceutical formulation and the lack of side effects, therefore, makes it an attractive candidate in human preventive care to reduce the risk of neurodegenerative diseases or during healthy aging. Other substances with antioxidant, anti-inflammatory and protective properties against mitochondria have recently been associated with PEA. Like PEA, these substances can cross the blood-brain barrier BBB and are also capable of inducing beneficial effects on the brain as regards neurodegeneration. For example, vitamin D (vitD) and α-lipoic acid (LA) have demonstrated their efficacy on the nervous system in physiological and pathological conditions and numerous pieces of evidence has been collected on their effects in neuronal aging [39,40,41,42,43,44,45,46]. VitD is synthesized in skin or ingested with food which has only one active form named vitamin D3 (vitD), cholecalciferol, or 1α,25 (OH) 2D3 [47]. This molecule regulates the expression through its specific receptor, vitamin D receptor (VDR), for many target genes, which control many cellular events including the protective role against oxidative stress, regulation of autophagic pathways [48], and interplay between apoptosis and survival pathways [49]. The antioxidant properties of LA include its ability to directly scavenge ROS, to regenerate endogenous antioxidants, such as glutathione and vitamins E and C, and to have a metal-chelating activity [50]. Even in its reduced form, the dihydrolipoic acid (DHLA) is considered an antioxidant compound [51]. Brain ageing has been also related to inflammation [52], induced by ROS that modulate cellular mechanisms for cell proliferation and survival, death, and immune responses by inducing the production of proinflammatory factors such as cytokines leading to cognitive dysfunctions and memory loss [40]. The experimental data of a research that studied the combination of vitD and LA have shown the high efficacy of these substances to keep cells viable in physiological or pathological conditions induced by H_2_O_2_. This combination was able to cross the BBB and remain in a very stable concentration to preserve cell viability. In this context, the aim of the study was to study a new PEA-based supplement combined with lipoic acid and vitamin D3 and prepared with a new technology (called FM-LipoMatrix^®^) in order to improve its absorption and crossing the BBB in order to modulate the mechanisms that lead to neuronal damages, such as neuroinflammation.

## 2. Materials and Methods

### 2.1. Agents Preparation

In both cell types, cells were treated with (0.1 µM) PEA dissolved in FM-LipoMatrix^®^ (a new technology based on solvent patent N°102017000036744 of noiVita srls, produced by Pro-Bio INTEGRA srl, Rovigo, Italy) in the presence or absence with lipoic acid (50 µM) plus vitamin D3 (100 nM) prepared directly into medium of stimulation (named LA + vitD) [39]. To verify the effectiveness of this new product the effects were compared to the classic commercial product (0.1 µM micronized PEA) and the solvent technology was also tested alone. The concentration of PEA was obtained by literature to maintain the antioxidant effect [53] and confirmed by dose-response study (data not shown) [54]. To verify the mechanism involved, some experiments were carried out also in presence of 30 min pre-treatments with both 10 µM AM251 and 10 µM AM630 (Cayman Chemical Company, Ann Arbor, MI, USA) [55], the specific CB1 and CB2 inhibitors, respectively. Finally, Lipopolysaccharides (LPS, Sigma-Aldrich, Milan, Italy) 500 ng/mL was used to verify the neuroprotective properties, pre-treating the cell for 24 h [56].

### 2.2. Astrocytes

Primary mouse astrocyte cultures were prepared from both male and female C57BL/6 mouse pups, following a standard technique described elsewhere [57] according to the National Guideline for the Use and Care of Laboratory Animals. Briefly, within 24 h of birth, pups were euthanized, and cortices were dissected and mechanically digested. The cell suspension was centrifuged at 800 rpm for 5  min. Pelleted cells were resuspended in Neuronal Basal Medium (Sigma-Aldrich, Milan, Italy), supplemented with 5% fetal bovine serum (FBS, Gibco, Waltham, Massachusetts, USA), 1% penicillin/streptomycin (Sigma-Aldrich, Milan, Italy), and 2  mM L-glutamine (Sigma-Aldrich, Milan, Italy), plated in multi-wells, and maintained in culture for 6 days before treatment. Astrocytes should be separated from microglia and oligodendrocyte precursor cells by shaking, as reported in literature [58]. For the experiments, 1 × 10^4^ cells on a 96-well plate were plated to study cell viability by the MTT test and ROS production by the colorimetric test; 1 × 10^6^ cells on a 6-well plate to analyze molecular pathways by western blot analysis.

### 2.3. Intestinal Barrier

The human intestinal Caco-2 cell line, purchased from American Type Culture Collection (ATCC, Manassas, VA, USA), was used as an experimental model [59] to predict the features of intestinal absorption following oral intake [60]. This cellular model is widely accepted to study bioavailability of drugs and xenobiotics, absorption, and metabolism. Furthermore, this cell line has been used in other studies on iron bioavailability [61]. These cells were grown in Dulbecco’s Modified Eagle’s Medium/Nutrient F-12 Ham (DMEM-F12, Sigma-Aldrich, Milan, Italy) containing 2 mM l-glutamine (Sigma-Aldrich, Milan, Italy), 1% penicillin-streptomycin (Sigma-Aldrich, Milan, Italy) and 10% fetal bovine serum (FBS, Sigma-Aldrich, Milan, Italy) at 37 °C in incubator at 5% CO_2_. Caco-2 cells, seeded on a transwell insert, were maintained in complete medium for 21 days [62,63] to induce differentiation of the cell, changing the culture medium every two days. After this time, cells were cultured under different pH conditions, with neutral pH (pH 7.4) in the basolateral chamber and acidic pH (pH 6.5) in the apical part [63,64]. Under these condition, PEA micronized, PEA FM alone and combined with lipoic acid and vitamin D3 were added to the apical environment in a time-course study (ranging from 1 h to 4 h), and, at each time point the intestinal absorption or bioavailability was detected by 0.04% fluorescein (sigma), a marker-dye applied to evaluate the transepithelial transport [65]. The amount of fluorescein transported was measured at 37 °C for 40 min by incubating Caco-2 cells in the absence or presence of PEA forms at the concentration reported above (apical pH, 6.0; basolateral pH, 7.4). The fluorescence was detected by a fluorescence spectrophotometer (multilabel plate reader, VICTOR X4, Perkin Elmer, Waltham, MA, USA) at 490/514 nm excitation/emission wavelengths. The results are expressed as the proportion of the original amount that permeated through the cells. The permeation rate [nmol min (mg protein)], J, was calculated following [65]:

J = J_max_ [C]/(K_t_ + [C]) (1)

where: C: the initial concentration of fluorescein. J_max_: the maximum permeation rate. K_t_: the Michaelis-Menten constant. Results are expressed as the means ± SD (%).

### 2.4. Cell Viability Test

MTT-based In Vitro Toxicology Assay Kit (Sigma-Aldrich, Milan, Italy) was performed on a 96-well plate to determine cell viability after each stimulation as previously described [64]. At the end of stimulation, cells were incubated with 1% MTT dye for 2 h at 37 °C in an incubator, 5% CO_2_, and 95% humidity, and then, the purple formazan crystals were dissolved in equal volume of MTT Solubilization Solution. Cell viability was determined using a spectrometer (VICTOR X4 multilabel plate reader, Perkin Elmer, Waltham, MA, USA) measuring the absorbance at 570 nm with correction at 690 nm and calculated by comparing results to the control.

### 2.5. ROS Production

The rate of superoxide anion release was used to examine ROS produced by astrocytes after stimulations [66]. In all samples (treated or not), 100 μL of cytochrome C was added after treatment and in another sample, for 30 min in an incubator was also added 100 μL superoxide dismutase (all substances were from Sigma-Aldrich, Milan, Italy). The absorbance was measured by a spectrometer (VICTOR X4 multilabel plate reader, Perkin Elmer, Waltham, MA, USA), at 550 nm, and O2 was expressed as of nanomoles per reduced cytochrome C per microgram of protein compared to the control on percentage (%).

### 2.6. Measurement of NO Production

Nitric oxide (NO) production by astrocyte was assessed by indirect measurement of nitrite concentration using Griess assay (Promega Corporation, Madison, WI, USA) following the manufacturer’s instruction, as previously described [67]. The absorbance was measured by a microplate reader (VICTOR X4 multilabel plate reader, Perkin Elmer, Waltham, MA, USA) at 550 nm. For calibration of the assay were used standards of sodium nitrite in range of 100–3125 μM (two-fold serial dilution).

### 2.7. MAPK Activity Assay

The measurement of phosphorylated ERK 1/2 in cell lysates were obtained using the InstantOneTM ELISA, following the manifacturer’s instructions (Thermo Fisher, Milan, Italy). Briefly, cells were lysed with 100 μL Cell Lysis Buffer Mix and 50 μL/well of each sample were tested in InstantOne ELISA microplate strips including the 50 μL/well Positive Control Cell Lysate and 50 μL/well negative control. At all wells 50 μL of prepared Antibody Cocktail were added and then the strips were incubated for 1h at room temperature on a microplate shaker, washed three times with 200 μL/well of Wash Buffer (1×), detected adding 100 μL of the Detection Reagent and stopped with 100 μL of Stop Solution. Using a spectrometer (VICTOR X4 multilabel plate reader, Perkin Elmer, Waltham, MA, USA) the absorbance was measured at 450 nm and results were expressed as means Absorbance (%) compared to control [49].

### 2.8. p53 Assay Kit

p53 activity was measured by p53 Transcription Factor assay kit (Cayman Chemical Company, Ann Arbor, MI, USA) on nuclear extracts following the manufacturer’s instructions. Briefly, the cells were lysed with ice-cold 1× Complete Hypotonic Buffer supplemented with NP-40, and then centrifuged at 12,000 *g* at 4 °C for 10 min. Pellet was solubilized with ice-cold Complete Nuclear Extraction Buffer 1x with protease and phosphatase inhibitors and then centrifuged at 12,000 *g* for 15 min at 4 °C; the supernatant was examined to analyze the activity of p53 related to the protein quantification through the BCA assay (Thermo Fisher, Milan, Italy) [40].

### 2.9. PPARα Assay Kit

PPARα was measured by PPAR alpha Transcription Factor Assay Kit (Abcam, Cambridge, UK) on nuclear extracts following the manufacturer’s instructions. Briefly, the cells were lysed with Nuclear Extraction Buffer supplemented with protease and phosphatase inhibitors and centrifuged at 14.000 *g* for 10 min at 4 °C. Complete Transcription Factor Binding Assay Buffer was added in supernatant that contains the nuclear fraction. Samples were added in plate and after addiction of PPAR alpha primary antibody and the second antibody, the absorbance was measured by a spectrometer (VICTOR X4 multilabel plate reader, Perkin Elmer, Waltham, MA, USA) at 450 nm and results were expressed as means Absorbance (%) compared to control.

### 2.10. CNR1 ELISA Test

CB1 receptors were quantified through the Mouse CNR1 ELISA Kit (My BioSource, San Diego, CA, USA), following the manifacturer’s instruction. Briefly, after stimulation cells were lysed by freeze cells at −20 °C and thaw with gentle mixing, repeating the freeze/thaw cycle for 3 times. At the end, the lysates were centrifuged at 3000 rpm for 15 min at 4 °C and the supernatants were collected and immediately used in assay kit. Samples were run in duplicate and all reagents were prepared immediately before use. The standard curve is used to determine the amount of CB1 in the sample measuring the optical density by absorbance at 450 nm (VICTOR X4 multilabel plate reader, Perkin Elmer, Waltham, MA, USA) and results expressed as means% compared to control values.

### 2.11. CNR2 ELISA Test

CB2 receptors were quantified through the Mouse CNR2 ELISA Kit (My BioSource, San Diego, CA, USA), following the manifacturer’s instruction based on sandwich enzyme-linked immune-sorbent assay technology. Briefly, after stimulation cells were lysed by freeze cells at −20 °C and thaw with gentle mixing, repeating the freeze/thaw cycle for 3 times. At the end, the lysates were centrifuged at 3500 rpm for 15 min at 4 °C and the supernatants were collected and immediately used in assay kit. Samples were run in duplicate and all reagents were prepared immediately before use. The standard curve is used to determine the amount of CB2 in the sample measuring the optical density by absorbance at 450 nm (VICTOR X4 multilabel plate reader, Perkin Elmer, Waltham, MA, USA) and results expressed as means% compared to control values.

### 2.12. NFKB Assay

Enzyme-Linked Immunosorbent Assay (ELISA) was carried out to analyze the NFKB DNA binding activity, following the manufacturer’s instruction (Cayman Chemical Company, Ann Arbor, MI, USA). Briefly, nuclear extracts were prepared using a nuclear extraction protocol [49] and NFKB contained in these extracts, was detected by addition of specific primary antibody. A secondary antibody conjugated to HRP is added to provide a sensitive colorimetric measured by a spectrometer (VICTOR X4 multilabel plate reader, Perkin Elmer, Waltham, MA, USA) at 450 nm and the concentration was calculated by comparing results to the standard curve.

### 2.13. Western Blot

Cells were lysed using ice Complete Tablet buffer (Roche, Basel, Swiss) supplemented with 1 mM phenylmethanesulfonyl fluoride (PMSF; Sigma-Aldrich, Milan, Italy), 1:100 mix Protease Inhibitor Cocktail (Sigma-Aldrich, Milan, Italy) and 2 mM sodium orthovanadate. From each lysate, 35 μg proteins was resolved into 8% and 15% SDS-PAGE gels, and polyvinylidene difluoride (PVDF) membranes (GE Healthcare Europe GmbH, Milan, Italy) were incubated overnight at 4 °C with a specific primary antibody: anti-CB1 (1:250, Santa Cruz, CA, USA), anti-CB2 (1:250, Santa Cruz, CA, USA), anti-ERβ (1:250, Santa Cruz, CA, USA), Annexin V (1:2000, Sigma, Milan, Italy). Protein expression was verified and normalized through β-actin detection (1:5000; Sigma, Milan, Italy).

### 2.14. Statistical Analysis

At least four independent experiments were run for each experimental protocol; the results are expressed as means ± SD of independent experiments performed on four technical replicates using One-way ANOVA followed by Bonferroni post hoc test for statistical analysis. *p* values < 0.05 were considered statistically significant.

## 3. Results

### 3.1. Time-Dependent Permeability on Caco-2 Cells Treated with PEA Alone and Combined

**Figure 1:**
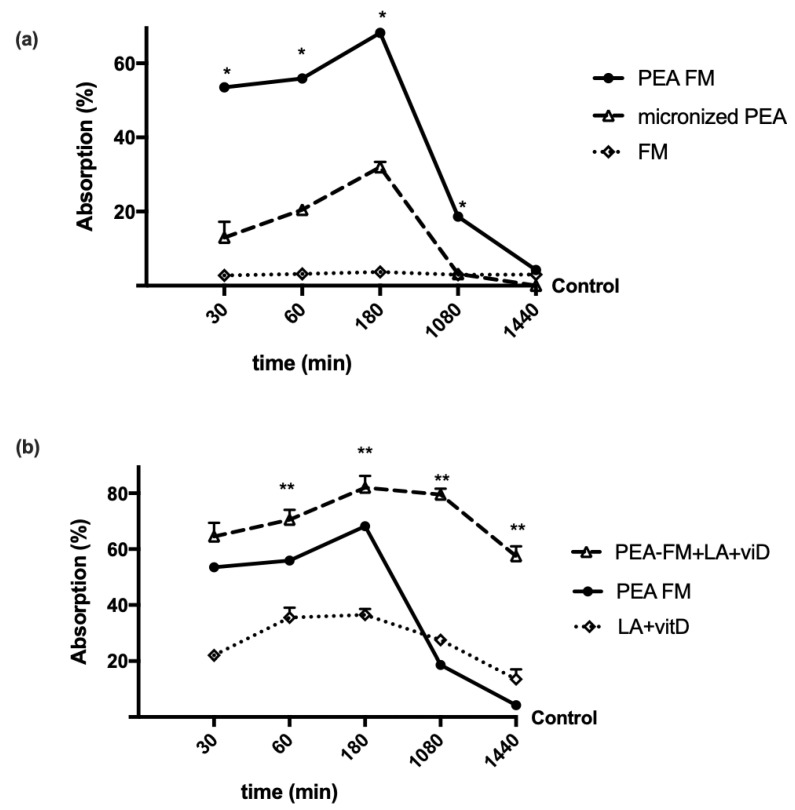
Intestinal absorption measured with fluorescein in a Caco-2 transwell system. PEA FM = PEA FM-LipoMatrix^®^; LA + vitD = lipoic acid plus vitamin D3; PEA FM + LA + vitD= PEA FM LipoMatrix^®^ plus lipoic acid and vitamin D3, FM = FM lipomatrix^®^ technique. Data are expressed as means ± SD (%) of five independent experiments normalized to control values. The results obtained from PEA FM are duplicated from panel (**a**) to (**b**) to improve the analysis of the data. PEA FM, micronized PEA, LA + vitD and PEA FM + LA + vitD *p* < 0.05 vs. control during all period analyzed. * *p* < 0.05 vs. micronized PEA, ** *p* < 0.05 vs. PEA FM.

To study the permeability characteristics of PEA-FM, some experiments were performed on Caco-2 in a transwell-based transporting set-up to evaluate its bioavailability compared to micronized PEA. The analysis of fluorescence at the basolateral environment, which evaluates the substance that crosses the intestinal membrane to enter the bloodstream, showed that both PEA forms had a time-dependent absorption (Figure 1a) starting from 30 min to 24 h compared to control (*p* < 0.05). In particular, the amount of PEA-FM was higher than micronized PEA for the entire period analyzed (*p* < 0.05), with a greater effect around 3 h, in which the absorption of PEA-FM was 120% compared to micronized PEA (*p* < 0.05), indicating that the permeability of PEA-FM was higher than that of micronized PEA during the intestinal emptying time (ranging from 1 h to 4 h). In addition, this better effect was related to the new technology FM which alone did not have significant effects (*p* < 0.05). In addition, as reported in Figure 1b, the effect of PEA-FM was also maintained in combination of LA + vitD compared to LA + vitD alone (*p* < 0.05) and to micronized PEA (*p* < 0.05) during all stimulation period. In particular, the main effect was observed starting from 3 h (*p* < 0.05); in particular main effect was observed at 3 h (about 20% compared to PEA-FM alone, about 156% to micronized PEA and about 125% to LA + vitD alone). These data confirmed previous findings about LA + vitD permeability and support the importance of PEA formulation to improve the bioavailability in order to improve its plasma concentration which is important to maintain a constant level to prevent a negative rebound effect during 24 h.

### 3.2. Direct Effect of Different PEA Forms on Astrocytes during Time

**Figure 2:**
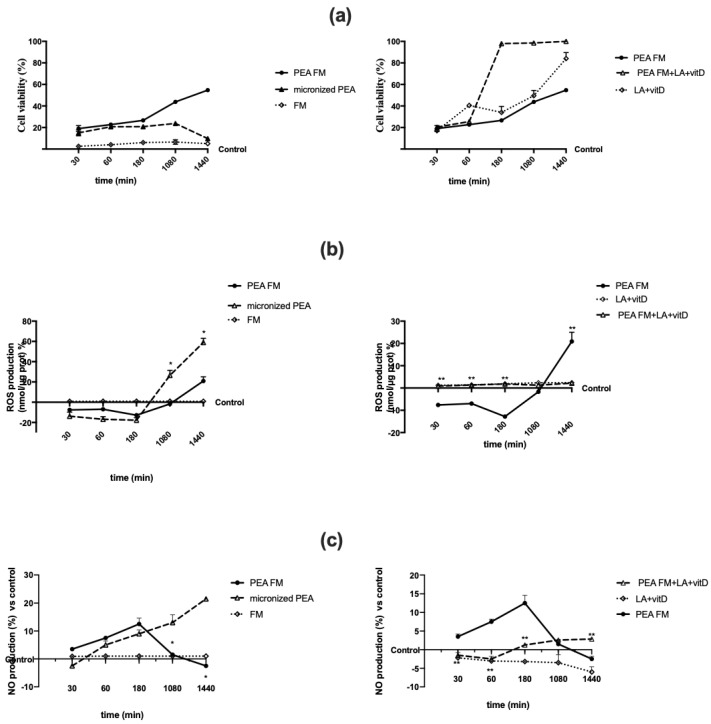
Cell viability, reactive oxygen species (ROS), and nitric oxide (NO) production in astrocytes. In (**a**) cell viability, in (**b**) ROS and in (**c**) NO production in astrocytes after stimulation. The abbreviations are the same as Figure 1. Data are expressed as means ± SD (%) of five independent experiments normalized to control values. The results obtained from PEA FM are duplicated between left and right graphs to permit the analysis among different groups of stimulation. In (**a**) all treatments *p* < 0.05 vs. control excluding FM stimulation alone. In (**b**) PEA FM 30, 60, 180, 1440 *p* < 0.05 vs. control. Micronized PEA: all stimulation time *p* < 0.05 vs. control. * *p* < 0.05 vs. micronized PEA, ** *p* < 0.05 vs. PEA FM. In (**c**) PEA FM: 60, 180 *p* < 0.05 vs. control, micronized PEA: 180, 1080, 1440 *p* < 0.05 vs. control. PEA FM + LA + vitD: 180, 1080, 1440 vs. control, * *p* < 0.05 vs. micronized PEA, ** *p* < 0.05 vs. PEA FM.

Since all substances used are well known to be able to cross BBB and to induce a beneficial effect on neuronal cells, the effectiveness of PEA-FM alone or combined with LA + vitD was tested on astrocytes analyzing cell viability, ROS and NO production in a time-course study. As reported in Figure 2a, cell viability was increased with both PEA forms starting from 30 min to 18 h compared to control (*p* < 0.05); in particular PEA FM showed a better increased during all period time compared to micronized PEA which was significant starting from 180 min (*p* < 0.05; about 33% compared to micronized at the same time) and to control (*p* < 0.05) also at 24 h (about 4 times compared to micronized at the same time). In addition, FM alone confirmed its no significant role. The combination of PEA FM + LA + vitD was able to amplify this effect; in particular the increase was amplified starting from 180 min (*p* < 0.05, about 270% compared to PEA FM) and this effect was maintained till to 24 h (*p* < 0.05, about 2 times compared to PEA FM). These findings demonstrate the importance of PEA formulation to induce a better influence on cell viability under physiological conditions and supporting the hypothesis of using PEA in combination with other neuroactive compounds to reduce neuronal vulnerability. In this context the same experiments were carried out to analyze ROS and NO production, to confirm the antioxidant and anti-inflammatory properties of PEA both alone and combined. As reported in Figure 2b, the time-course study confirmed the antioxidant properties of both PEA forms within 3 h of stimulation. However, PEA FM was able to induce a significant ROS reduction compared to micronized PEA starting from 3 h to 24 h of stimulation (*p* < 0.05) supporting the hypothesis of no rebound effect. In addition, the presence of LA + vitD with PEA FM amplified this effect and ROS production was maintained like control values (*p* > 0.05) during all time of stimulation. Finally, the NO release (Figure 2c) revealed similar results to what obtained on ROS analysis, indicating the balance of mitochondrial activity and of antioxidant/anti-inflammatory; in particular, the best results were obtained by PEA FM combined with LA + vitD. All these data support the hypothesis of a possible new strategy to restore and prevent brain function to slow down brain aging in humans.

### 3.3. Molecular Mechanism Activated by Different PEA Forms on Astrocytes

**Figure 3:**
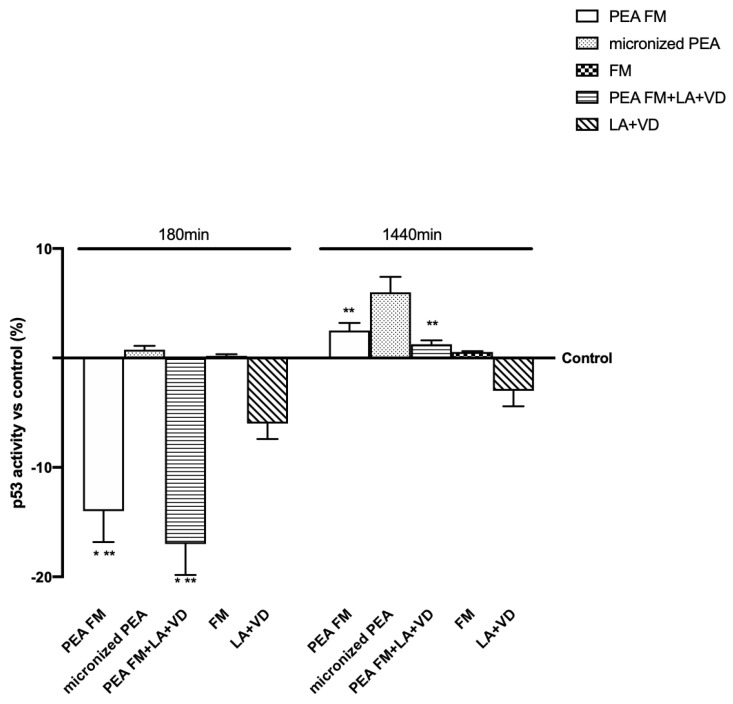
p53 activity in astrocytes after stimulation. The abbreviations are the same in Figure 1. Data are expressed as means ± SD (%) of five independent experiments normalized to control values. * *p* < 0.05 vs. control, ** *p* < 0.05 vs. micronized PEA.

Since previous findings demonstrated the ability of PEA to induce beneficial effects on the nervous tissue, another important point that needs to be investigated includes the mechanism activated on astrocytes. It was considered very important to verify the cell integrity through p53 activation and the involvement of PPARα and endocannabinoids receptors (CB1 and 2) leading to MAPK/ERKs activation. Moreover, the time of stimulation at 180 min and 1440 min was chosen to verify the mechanism at the maximum effects previously observed and at the end of that. As reported in Figure 3, p53 activity was maintained under the control level by all formulations tested; in particular micronized PEA, with or without vitD and LA, exerted better effects than other compounds at 180 min, confirming the safety of FM technology on this cell type.

**Figure 4:**
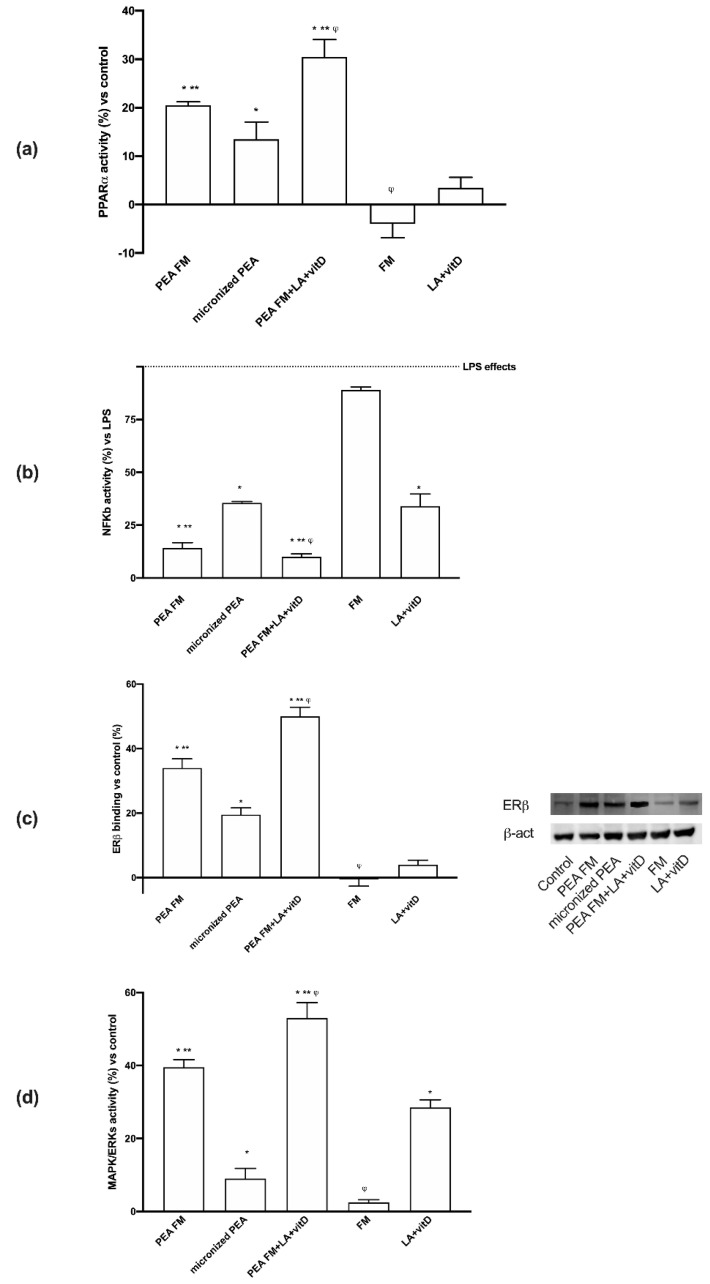
Western blot, densitometric analysis and molecular activity. In (**a**) (**b**) and (**d**) PPARα, NFKB activation and ERK activity respectively and in (**c**) ERβ analyzed by Western blot and densitometric analysis. The abbreviations are the same in Figure 1. Data are expressed as means ± SD (%) of five independent experiments normalized to control values. * *p*<0.05 vs. control, ** *p*< 0.05 vs. micronized PEA, ^φ^ *p* < 0.05 vs. PEA FM. In (**b**) LPS = lipopolysaccharides. All samples are significant to control values. * *p* < 0.05 vs. LPS; ** *p* < 0.05 vs. micronized PEA; ^φ^ *p* < 0.05 vs. PEA FM.

As regards the mechanisms activated, both PPARα and ECs were involved; indeed, as shown in Figure 4a, PEA FM was able to induce PPARα with more efficiency compared (*p* < 0.05) to micronized PEA (about 52% at 24 h), but FM alone was unable. These data confirm previous results about the importance of PEA preparation to induce a beneficial effect. In addition, the combination with LA + vitD demonstrates the ability to amplify this effect (*p* < 0.05) at 24 h (about 51%) compared to PEA FM (*p* < 0.05), indicating a positive influence of LA + vitD on the anti-inflammatory mechanisms. In addition, some experiments were carried out in presence of 500 ng/mL LPS to verify the ability of the substances to have neuroprotective properties. As reported in Figure 4b, LPS was able to induce NFKB activation (*p* < 0.05), confirming data present in literature. The successive stimulation with PEA FM reduced this activation about 85% (*p* < 0.05) and this effect was better than micronized PEA which reduces NFKB activation about 64% (*p* < 0.05). The combination with PEA FM and LA + vitD improves this beneficial effect reducing about 90% NFKB activation caused by LPS conditioning. These results confirmed the ability of PEA FM alone and combined with LA + vitD to reduce neuroinflammatory markers involved both on neurodegenerative condition and neuronal aging.

As regards another important possible mechanism activated by PEA to modulate neuroinflammation, the role of ERβ was also investigated. As shown in Figure 4c, PEA FM was able to bind ERβ with more effectiveness (*p* < 0.05) compared to micronized PEA (about 70%) and this effect was amplified by the presence of LA + vitD (*p* < 0.05) compared to PEA FM alone (about 48%) and to micronized PEA (about 150%). These findings are important to support the possible use of both PEA FM alone or combined with LA + vitD in humans because ERβ plays a major role in providing nutrient supply for neurons and regulating systemic metabolism and energy balance.

To complete the main markers useful by PEA to induce beneficial effects, ERK/MAPK activity was investigated. As illustrated in Figure 4d, PEA FM induced a major effect on ERK activation compared to micronized PEA (about 300%, *p* < 0.05) and the presence of LA + vitD amplified this effect (about 32% compared to PEA FM alone and about five times compared to micronized PEA). Based on all these findings, it can be stated that PEA FM combined with lipoic acid and vitamin D3 has a great effectiveness in preventing age-dependent brain damage by acting with anti-inflammatory and antioxidant mechanisms. This data suggest that the neuronal vulnerability may be reduced by the habitual use of the combined product. In addition, it confirms the importance of the tested formulation to induce its beneficial effects, as reported by the comparison between PEA FM and micronized PEA.

**Figure 5:**
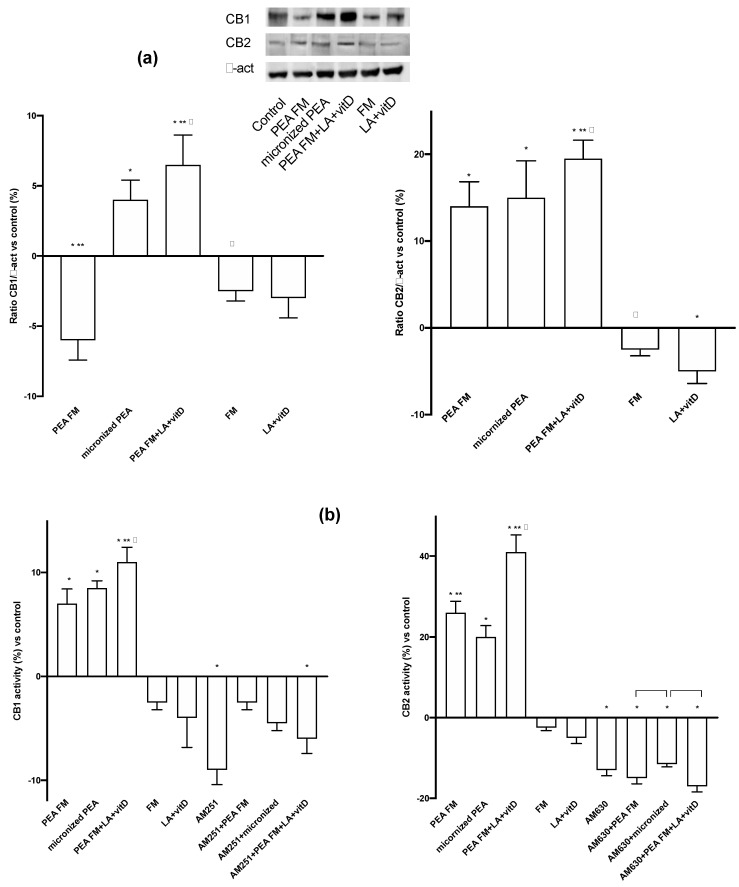
Western blot, densitometric analysis and activity of CBs receptors. In (**a**) western blot and densitometric analysis of CB1 and CB2 receptors; in (**b**) analysis of CB1 and CB2 activity in presence or absence of specific blockers, AM251 and AM630, respectively. The abbreviations are the same in Figure 1. Data are expressed as means ± SD (%) of five independent experiments normalized to control values. * *p* < 0.05 vs. control, ** *p* < 0.05 vs. micronized PEA, ^φ^ *p* < 0.05 vs. PEA FM, the bars *p* < 0.05 between the treatments with blockers.

The involvement of both CB1 and CB2 receptors are also confirmed (Figure 5a) at 24 h. During experiments, PEA FM was able to act through CB2 receptor (13% vs. control) with similar effect to micronized PEA (about 15% vs. control), but was unable to induce the CB1 activation contrary to what demonstrated by micronized PEA (*p* < 0.05). On the contrary, the presence of LA + vitD with PEA FM increased the CB2 activation and involved also the CB1 and on both receptors this combination acted better than micronized PEA, indicating that this is the best combination capable of stimulating the endocannabinoid receptors; in detail, acting both on the vulnerability mechanisms controlled by CB1 and on the prevention of neurodegeneration by CB2. To verify these qualitative tests, two ELISA assays were performed also in presence of the specific CB1 and 2 blockers, AM251 and AM630, respectively. As reported in Figure 5b, the absence of the blockers confirmed the data obtained from western blot; in particular the main activation of CB2 was observed and the main effect was obtained by the PEA FM combined with LA + vitD. On the contrary, the presence of the blockers inhibits all these effects only in presence of AM630, confirming the ability PEA to act with main affinity to CB2 receptor.

## 4. Discussion

Chronic pain affects many people in the Western world, constituting an enormous burden for the individuals and society [68]. Current analgesics are mainly based on molecules that reduce pain perception, transduction, transmission, and modulation in neurons and/or reduce peripheral inflammation. The nature of these pharmacological targets is likely to be the principal cause of their limited success in controlling disease progression [69]. Inflammation is an important cellular defence mechanism aimed toward at removing injuries stimuli and initiating the healing process. However, when prolonged, it can override the bounds of physiological control and becomes destructive. Inflammation is a key element in the pathobiology of chronic pain, neurodegenerative diseases, stroke, spinal cord injury, and neuropsychiatric disorders [15]. Chronic inflammatory processes could be blocked by a resolution program which includes the lipid mediator’s production endowed with the capacity to switch off inflammation. These naturally occurring lipid signaling molecules that include the N-acylethanolamines, N-arachidonoylethanolamine (an endocannabinoid), and its congener N-palmitoylethanolamine (palmitoylethanolamide or PEA) [15]. PEA is especially given in humans for its analgesic and anti-inflammatory characteristics and has displayed high safety and tolerability.

Compounds that are present in nature, introduced through food, can therefore play an important role in maintaining the well-being of everyone. Many preclinical in vitro and in vivo studies have demonstrated PEA acts on several molecular targets to induce its biological activity in both central and peripheral nervous systems. These multiple mechanisms of action clearly differentiate PEA from classic anti-inflammatory drugs and are attributed to the compound that has quite unique anti(neuro)inflammatory properties [70]. PEA may have a role in modulating cellular homeostasis when it interacts with external stressors provoking, for example, inflammation. PEA is capable to induce biological effects in mast cell-mediated models of neurogenic inflammation and neuropathic pain and is neuroprotective property in models of stroke, spinal cord injury, traumatic brain injury, and Parkinson disease. PEA in micronized/ultramicronized form demonstrates its superior oral efficacy in inflammatory pain models when compared to naïve PEA. Inhibiting or modulating the enzymatic breakdown of PEA could be a complementary therapeutic approach to treat neuroinflammation. The anti-inflammatory action of PEA combined with other molecule could potentiate its pharmacological effects [1].

The possibility through a healthy diet aimed at reinforcing our immune system, for example by enhancing the action of endogenous molecules, such as LA, vitD, and, in particular, PEA, can be a valid alternative to the use of anti-inflammatory drugs, or a possible therapy that works in synergy with the latter [71].

In this article 3 different formulation were tested: PEA micronized, FM-LipoMatrix^®^, an innovative technology which improves the bioavailability of lipophilic bioactive molecules and FM-LipoMatrix^®^ supplemented with vitD and LA.

Given their lipidic nature and enormous particle size in the native state, molecules like PEA may have limitations in terms of solubility and bioavailability. The use of micronization for dissolution enhancement of poorly water-soluble drugs is a technique frequently used in the pharmaceutical field [72].

In this study was demonstrated that the association of FM-LipoMatrix^®^ with vitD and LA increased permeability and support the importance of PEA formulation to improve the bioavailability in order to improve its plasma concentration which is important to maintain in constant level to prevent the negative rebound effect during 24 h.

Since these substances are well known to be able to cross BBB barrier and to induce a beneficial effect on neuronal cells, the efficacy of different formulation of PEA were tested on astrocytes investigating cell viability, ROS, and NO productions. Combination of FM-LipoMatrix^®^, vitD and LA showed a better effect in increasing cell viability supporting the hypothesis to use PEA in combination with other brain molecules to modulate neuronal aging. To confirm the antioxidant and anti-inflammatory properties of PEA, FM-LipoMatrix^®^ with vitD and LA showed a reduction in ROS production, supporting the hypothesis of no rebound effect. In addition, as regards NO production this formulation was able to maintain NO level like control value indicating the balance of mitochondrial activity and of antioxidant/anti-inflammatory.

Another important point to verify was the integrity of cells; indeed, p53 level after stimulation with different PEA formulation was maintained under control level. FM-LipoMatrix^®^ with vitD and LA showed a better reduction during time.

The molecular target through which PEA plays its anti-inflammatory, analgesic, and neuroprotective role is PPARα [73] and through several indirect mechanisms, that includes the endocannabinoid-mediated activation of CB1 and CB2 receptors [3,74].

PEA FM was able to increase PPARα level, but the combination of FM-LipoMatrix^®^, vitD and LA was able to induce PPARα with more efficiency than the other formulations, indicating that the association with vitD and LA amplify this effect. These findings demonstrate that combination with FM-LipoMatrix^®^ acts a ligand of PPARα, might regulate neurosteroidogenesis modulating neuroinflammation.

In addition, the involvement of both CB1 and CB2 receptors were also confirmed. PEA FM was able to act through CB2 receptor with similar effect to PEA micronized but was not able to induce the CB1 activation. On the contrary, the combination of FM-LipoMatrix^®^, vitD and LA increased the CB2 activation and induce also the CB1 and on both receptors acts better then PEA micronized, indicating that this combination was able to act with a greater effectiveness on endocannabinoid receptors, in particular acting both on anti-aging defence by CB1 and on preventing neurodegeneration by CB2.

Although it is well documented that activation of either ERα or ERβ has a significant neuroprotection, with the involvement of MAPK pathway, which is an important part of neuroprotective mechanism [75]. Our results are important to support the idea that ERα and ERβ, which are found in nuclear, cytoplasmic and membrane sites throughout the brain, can regulate metabolism perhaps by acting as a leptin mimetic in the central nervous system (CNS) [75]. In this context, the ability of PEA to act by these ways may be important to predict additional functional of PEA such as on neuronal aging. We can affirm that PEA FM combined with lipoic acid and vitamin D3 has greater efficacy in preventing age-dependent brain damage by acting with anti-inflammatory and antioxidant mechanisms and neuronal vulnerability may be reduced by the usual use of the combined product.

The development of novel formulations of PEA or the combination with vitD and LA has improved the efficacy of PEA. Moreover, PEA was avoided of adverse reactions worthy of notes in clinical studies. Preclinical and clinical studies about PEA efficacy in neurological disease report encouraging outcomes and are considerably multiplying in these last years, which is already a clear sign of its enormous therapeutic potential.

## 5. Conclusions

PEA FM plus LA + VD is a novel formulation of PEA which used a new technology, FM-LipoMatrix^®^, as a carrier to improve the absorption of PEA which have two important brain co-modulator useful to amplify the beneficial effects on ageing neuronal cells. Basing on results described above on PEA FM + LA + VD, this in vitro study supports some important beneficial effects including decreases pain sensitivity, improves locomotor function, reduces inflammatory signs and mediators, and lowers histological damage score than other PEA forms. PEA shows considerable versatility in counteracting inflammation with the new formulation based on PEA-FM. The possibility of using PEA in association with other natural molecules, such as LA and vitD, demonstrates that PEA can act effectively not only individually, but also and above all in synergy with other molecules. This additional property may be important to amplify the range of use in human diseases.
